# Change in abdominal obesity after colon cancer surgery – effects of left-sided and right-sided colonic resection

**DOI:** 10.1038/s41366-023-01445-8

**Published:** 2024-01-03

**Authors:** Younes Kays Mohammed Ali, Troels Gammeltoft Dolin, Janus Damm Nybing, Jakob Lykke, Frederik Hvid Linden, Erik Høgh-Schmidt, Thorkild I. A. Sørensen, Jesper Frank Christensen, Yousef J. W. Nielsen, Jim Stenfatt Larsen, Sten Madsbad, Julia Sidenius Johansen, Maria Saur Svane, Louise Lang Lehrskov

**Affiliations:** 1 Department of Endocrinological Research, Copenhagen University Hospital –Amager and Hvidovre, Hvidovre, Denmark; 2 Department of Medicine, Faculty of Health and Medical Sciences, University of Copenhagen, Copenhagen, Denmark; 3 Department of Medicine, Copenhagen University Hospital – Herlev and Gentofte, Herlev, Denmark; 4 CopenAge, Copenhagen Center for Clinical Age Research, University of Copenhagen, Copenhagen, Denmark; 5 Department of Radiology, Copenhagen University Hospital – Bispebjerg, Copenhagen, Denmark; 6 Department of Surgery, Copenhagen University Hospital – Herlev and Gentofte, Herlev, Denmark; 7 Novo Nordisk Foundation Center for Basic Metabolic Research and Department of Public Health, University of Copenhagen, Copenhagen, Denmark; 8 Centre for Physical Activity Research, Copenhagen University Hospital – Rigshospitalet, Copenhagen, Denmark; 9 Department of Sports Science and Clinical Biomechanics, Faculty of Health Sciences at the University of Southern Denmark, Odense, Denmark; 10 Digestive Disease Center, Bispebjerg Hospital, Copenhagen, Denmark; 11 Department of Radiology, Copenhagen University Hospital – Herlev and Gentofte, Herlev, Denmark; 12 Department of Oncology, Copenhagen University Hospital – Herlev and Gentofte, Herlev, Denmark; 13 Department of Gastrointestinal Surgery, Copenhagen University Hospital Amager and Hvidovre, Hvidovre, Denmark

**Keywords:** Endocrinology, Obesity, Metabolic syndrome

## Abstract

**Background:**

Excess abdominal visceral adipose tissue (VAT) is associated with metabolic diseases and poor survival in colon cancer (CC). We assessed the impact of different types of CC surgery on changes in abdominal fat depots.

**Material and methods:**

Computed tomography (CT)-scans performed preoperative and 3 years after CC surgery were analyzed at L3-level for VAT, subcutaneous adipose tissue (SAT) and total adipose tissue (TAT) areas. We assessed changes in VAT, SAT, TAT and VAT/SAT ratio after 3 years and compared the changes between patients who had undergone left-sided and right-sided colonic resection in the total population and in men and women separately.

**Results:**

A total of 134 patients with stage I-III CC undergoing cancer surgery were included. Patients who had undergone left-sided colonic resection had after 3 years follow-up a 5% (95% CI: 2–9%, *p* < 0.01) increase in abdominal VAT, a 4% (95% CI: 2–6%, *p* < 0.001) increase in SAT and a 5% increase (95% CI: 2–7%, *p* < 0.01) in TAT. Patients who had undergone right-sided colonic resection had no change in VAT, but a 6% (95% CI: 4–9%, *p* < 0.001) increase in SAT and a 4% (95% CI: 1–7%, *p* < 0.01) increase in TAT after 3 years. Stratified by sex, only males undergoing left-sided colonic resection had a significant VAT increase of 6% (95% CI: 2–10%, *p* < 0.01) after 3 years.

**Conclusion:**

After 3 years follow-up survivors of CC accumulated abdominal adipose tissue. Notably, those who underwent left-sided colonic resection had increased VAT and SAT, whereas those who underwent right-sided colonic resection demonstrated solely increased SAT.

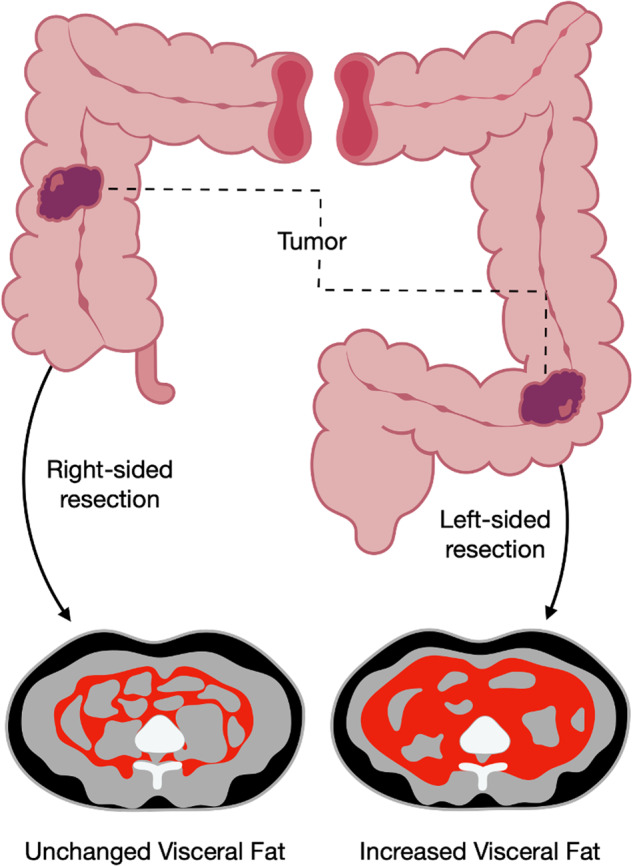

## Introduction

Colon cancer (CC) is the fourth most common cancer worldwide [1] and it has a still increasing incidence rate [1, 2]. Survival after a CC diagnosis is steadily improving due to early detection, diagnostic precision and more advanced cancer treatment, and today more than 70% of patients with non-metastatic CC are alive after 5 years [3]. Unfortunately, the risk of developing metabolic comorbidities such as obesity, type 2 diabetes (T2D) and cardiovascular disease (CVD) after a CC diagnosis is also increasing [4, 5] with worsening of overall and cancer-specific prognosis in these patients [6].

Obesity and especially excess abdominal fat are factors increasing morbidity [7] and mortality [8] in survivors of CC. The total amount of abdominal adipose tissue (TAT) consists of the visceral adipose tissue (VAT) and the subcutaneous adipose tissue (SAT) [9]. Distribution of body fat varies between men and women [10]. Men typically have a higher amount of VAT [10]. The structure and functions of the two tissues are very different [11] with VAT being highly pro-inflammatory and a major risk factor of developing several diseases including T2D [12], CVD [13] and various cancer types [14, 15]. Excess VAT is associated with CC development [16] and a meta-analysis from 2014 showed a linear association between CT-assessed VAT area and adenomas, a CC precursor [17]. Moreover, excess VAT may increase CC relapse [18]. In contrast excess SAT hasn’t been associated with worse outcomes of CC [19].

The ratio of VAT/SAT (V/S) can be used as a prognostic marker, and a high V/S ratio has been associated with an increased risk of metabolic disturbances in a general population and with cancer recurrence in patients with CC [20, 21]. In contrast, weight loss, reducing VAT, decrease the risk of developing diseases such as T2D [22], CVD [23] in obese populations and improve the prognosis of CC [24]. We and others have shown that exercise training effectively reduces abdominal VAT in subject with overweight [25], which supports the importance of exercise training in survivors of cancer.

We recently found a post-treatment increase in VAT in survivors of colorectal cancer who were colonic resected and speculated whether the surgical treatment per se may affect metabolism including fat deposits in the body [26].

Two clinical-epidemiological studies revealed that patients treated with left-sided compared with right-sided colonic resection for CC and other colonic illnesses had an increased risk of developing T2D and CVD respectively [27, 28]. These findings raise the intriguing question whether resection of CC, and especially left-sided colonic resections, contribute to metabolic disturbances in survivors of CC.

Due to the significant impact of excess abdominal fat on CC prognosis, it may be of great clinical relevance to identify survivors of CC with a particular risk of VAT accumulation in order to initiate preventive strategies or treatment. Despite the association between excess VAT and CC prognosis no specific follow up regimes or interventions are offered to overweight survivors of CC.

The primary aim of this study was to investigate changes in abdominal VAT, SAT, TAT and V/S ratio after 3 years in patients who had undergone left-sided compared with right-sided colonic resection as part of CC treatment. Secondary aim was to examine changes over time in VAT, SAT, TAT and in V/S ratio after both CC resection types. Moreover, we aimed to examine the changes described above when stratifying subjects by sex.

## Methods

### Study design and population

We conducted a historical prospective study of survivors of stage I-III CC, who had undergone intended curative cancer treatment with right-sided colonic resection (right-sided hemicolectomy) or left-sided colonic resection (left-sided hemicolectomy, sigmoid resection) between 2014–2018 at Department of Surgical Gastroenterology at Copenhagen University Hospital – Herlev and Gentofte, Herlev, Denmark. Following cancer surgery some patients, depending on cancer stage, were treated with 3–6 months of adjuvant chemotherapy (FOLFOX (5-fluorouracil (5-FU), leucovorin, and oxaliplatin), CAPOX (capecitabine and oxaliplatin), monotherapy 5-FU or capecitabine) [29, 30]. As prophylaxis of acute nausea and vomiting, prednisolone was administered for a few days when a new cycle of chemotherapy was initiated [31]. In this study 50 mg/day prednisolone was administered on the day of treatment and 25 mg/day the following 2 days every second or third week, depending on the applied chemotherapy regime. However, this only applies to patients treated with FOLFOX or CAPOX, if patients were treated with monotherapy 5-FU or capecitabin no prednisolone was administered. Thus, some patients treated with adjuvant chemotherapy may have received up to 6 days of prednisolone monthly for up to 6 months.

**Fig. 1:**
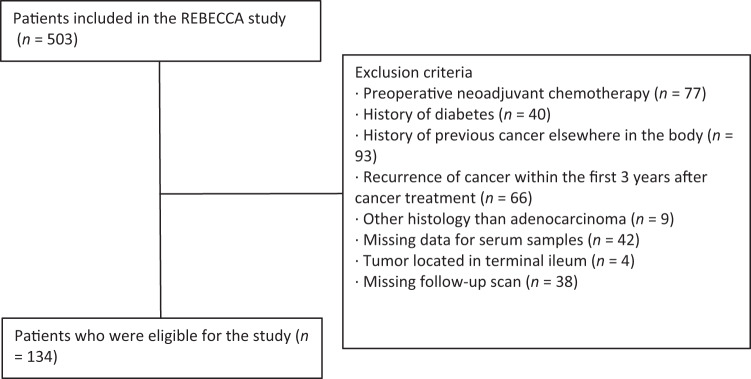
Flowchart.

All patients were included prior to cancer resection in the Danish REBECCA study (“Biomarkers in patients with colorectal cancer – can they provide new information on the diagnosis, treatment efficacy, adverse effects and prognosis?”), an ongoing cohort study initiated in July 2014. The cohort and study design have been described in detail previously [32]. The current study is a subgroup analysis of patients that did not meet the exclusion criteria: UICC-stage IV tumors, rectal cancer (the border between the sigmoid colon and the rectum was defined as 15 cm beyond the anal verge), active cancer at any site during the 3 year postoperative follow-up period, previous colonic resection, a history of diabetes (including current use of diabetes medication), cases where a stoma was performed peri-operatively or recurrence of cancer within the first 3 years after cancer treatment. A total of 134 patients with CC were eligible for the study and were included in the analysis (Fig. 1).

All patients underwent abdominal CT-scans preoperatively for disease staging, which was repeated after 3 years according to the surveillance program to monitor cancer relapse [33]. Medical records were reviewed for all patients and information on age, sex, height, weight, ECOG performance status, lifestyle comorbidities (chronic obstructive pulmonary disease, hypertension, dyslipidemia or cardiovascular disorders), antihypertensive or steroid medication, tobacco and alcohol consumption were collected preoperatively as well as tumor location in the colon, date of the CT-scans (preoperatively and 3-years postoperatively), date of surgery, potential postoperative adjuvant treatment, peri- or postoperative complications to surgery and development of diabetes during the 3 years of surveillance. Additionally, medication lists were reviewed on a common medication server to make sure that outpatients diagnosed and treated with antidiabetic medication outside of hospitals were revealed. Clinical data were collected blinded to VAT and SAT measurements.

### CT-scans

**Fig. 2:**
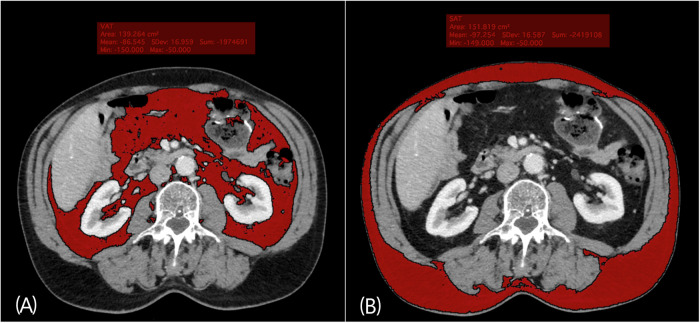
Determination of abdominal adipose tissue area by quantitative CT-scan. The analysis was performed at L3-level at the point where both transversal processes were visible. Adipose tissue areas were indicated by Hounsfield scale between −150 and −50 HU, which is an attenuation value specific for fatty tissues. Red color marks (**A**) VAT-area (**B**) SAT-area. Direction of scanning view: Top-down.

VAT and SAT areas were measured using venous phase axial CT images at the L3-level of the lumbar spine, using the exact axial image where both transversal processes were visible. We used the open-source code software Horos™ (version 3.3.6., Annapolis, MD USA) to define the adipose tissue area, indicated by tissue with Hounsfield units (HU) between −150 and −50 HU (Fig. 2), which is an attenuation value specific for fatty tissues [34]. We defined VAT as the compartment limited anteriorly and laterally by the abdominal musculature and posteriorly by the vertebral column and paraspinal musculature as previously described [35]. SAT was defined as adipose tissue located external to the abdominal and back musculature. Total adipose tissue (TAT) was calculated as the sum of the two areas. The use of CT images to determine abdominal fat depots is widely used [36] and images at L3-level of the lumbar spine has been shown to offer the highest prediction of total SAT and VAT depots in the body compared with CT images at other levels of the lumbar spine [37].

The VAT/SAT-ratios (V/S ratio) were calculated by dividing the mean VAT-area with the mean SAT-area.

All analyses were performed blinded to type of surgery (right- or left-sided resection) and were performed by two independent investigators, one of them being a radiographer. The intraclass correlation was determined and in case of discordance >5% between the investigators, both investigators re-evaluated their analyses. The average adipose tissue area measured by the two investigators was used in subsequent statistical analyses.

### Biomarker analysis

Blood was drawn just prior to planned cancer surgery. C-reactive protein (CRP) was consecutively measured as part of the clinical blood work using high-sensitive CRP ultra-ready-to-use, liquid assay reagent by an immunoturbidimetric method on a fully automated chemistry analyzer (Kit-test SENTINEL CRP Ultra (UD), 11508 UD-2.0/02 2015/09/23) at the Department of Clinical Biochemistry. The blood samples were centrifuged at 2300 g at 4 °C for 10 min, and serum was then aliquoted and stored at −80 °C. Interleukin-6 (IL-6) was measured using a high-sensitive enzyme-linked immunosorbent assay (ELISA) (Quantikine HS600B, R&D Systems, Abingdon, UK) in accordance with the manufacturer’s instructions. IL-6 was measured using the same ELISA batch.

### Statistical analyses

The primary analysis compared difference in changes in VAT, SAT, TAT and V/S ratio preoperatively to 3 years after cancer surgery. The primary analysis was based on a mixed effect model using data from all patients and performed with time, group and preoperative values as fixed effects, individual subjects as random effects and the ‘time x group’ interaction was the primary readout. For all study outcomes, the raw means and standard errors of the means (SEMs) were reported for each group for the preoperative and 3 years timepoint, along with the estimated in-group changes and between-group changes with 95% confidence intervals (CI) extracted from the mixed effects model. All variables were log-transformed to improve model compliance, accordingly the estimated in-group changes and between-group difference were analyzed on a log-scale and were reported as back-transformed relative ratios with 95% CIs. As the specified mean changes are based on estimates from the mixed effects models it might not reflect the numerical change. Thus, for example a back-transformed estimate of 0.95 corresponds to a median relative change of −5%. Differences in baseline characteristics between the right-sided and left-sided resected patients preoperatively were analyzed using unpaired *t*-tests. Results were considered statistically significant if *p* < 0.05. All the statistical analyses were performed using R-software (version 1.3.1056).

### Ethics statement

This study is conducted in accordance with the Declaration of Helsinki. All experimental protocols (The REBECCA study protocol (VEK j.nr. H-2-2013-078) and additional protocol for current project (78485)) were approved by “The ethical committee of the capital region of Denmark” and the Danish Data Protection Agency in Copenhagen, Denmark (j. No. HEH-2014-044, I-suite No. 02771 and PACTIUS P-2019-614). Informed consent was obtained from all subjects included in the REBECCA study.

## Results

### Baseline characteristics

Preoperative baseline characteristics of the 134 patients with stage I-III CC were similar in the two resection groups (Table 1). Although not statistically significant, patients who had undergone left-sided resection (*n* = 80) were more likely men, had a better ECOG performance status and had more likely completed chemotherapy than patients who had undergone right-sided resection (*n* = 54). The cancer stage was similar in the groups except from stage 3b, which was more common in the group of left-sided resections. The higher cancer stage in this group resulted in a significantly more frequent treatment with adjuvant chemotherapy in patients who had undergone left-sided resection compared to patients who had undergone right-sided resection (left-sided: 41,3%; right-sided: 25,9%; *p* < 0.01).

**Table 1:** Baseline characteristics for all patients.

	All *n* = 134	Left sided resection *n* = 80	Right sided resection *n* = 54	*p*-value
Sex, *n* (%)				
Male	62 (46.3)	40 (50.0)	22 (40.7)	0.07
Female	72 (53.7)	40 (50.0)	32 (59.3)	0.07
Age, year, mean (SD)	67 (8.9)	66 (8.7)	69 (9.0)	0.06
Overall stage, *n* (%)				
1	38 (28.4)	21 (26.3)	17 (31.5)	0.52
2a	51 (38.1)	29 (36.3)	22 (40.7)	0.60
2b	5 (3.7)	3 (3.8)	2 (3.7)	0.99
3a	12 (9.0)	5 (6.3)	7 13.0)	0.21
3b	28 (20.9)	22 (27.5)	6 (11.1)	0.01
ECOG performance status, *n* (%)				
0	111 (82.8)	70 (87.5)	41 (75.9)	0.10
1	17 (12.7)	9 (11.3)	8 (14.8)	0.56
2	6 (4.5)	1 (1.3)	5 (9.3)	0.06
Anthropometrics, mean (SD)				
Weight, kg	78.4 (15.7)	79.6 (15.8)	76.6 (15.6)	0.29
Height, cm	172.5 (9.8)	172.4 (9.8)	172.6 (9.9)	0.91
BMI, kg/m^2^	26.3 (4.6)	26.8 (4.8)	25.6 (4.2)	0.16
Smoking, *n* (%)				
Never	64 (47.8)	40 (50.0)	24 (44.4)	0.53
Former	51 (38.1)	31 (38.8)	20 (37.0)	0.84
Current	14 (10.4)	5 (6.3)	9 (16.7)	0.08
Not known	5 (3.7)	4 (5.0)	1 (1.9)	0.50
Alcohol, *n* (%)				
Never	32 (23.9)	21 (26.3)	11 (20.4)	0.43
Normal	78 (58.2)	45 (56.3)	33 (61.1)	0.58
Overuse	20 (14.9)	11 (13.8)	9 (16.7)	0.65
Previous overuse	1 (0.7)	1 (1.3)	0 (0.0)	0.32
Not known	3 (2.2)	2 (2.5)	1 (1.9)	0.8
Comorbidities, *n* (%)				
Hypertension	44 (32.8)	25 (31.3)	19 (35.2)	0.64
Hyperlipidemia	29 (21.6)	16 (20.0)	13 (24.1)	0.58
COPD	10 (7.5)	5 (6.3)	5 (9.3)	0.53
Cardiovascular disease	8 (6.0)	4 (5.0)	4 (7.4)	0.58
Others	3 (2.2)	2 (3)	1 (1.9)	0.8
Pharmacological treatment, *n* (%)				
Prednisolone	4 (3.0)	2 (3.0)	2 (3.7)	0.70
Antihypertensive medication	89 (66.4)	51 (63.8)	38 (70.4)	0.43
Biochemical values, median (range)				
IL6, ng/L	2.2 (0.6–71.9)	2.0 (0.6–59.6)	2.4 (0.9–71.9)	0.47
CRP, mg/L	1.0 (0.0–76.0)	0.8 (0.0–76.0)	1.0 (0.0–51.9)	0.86

Postoperatively only around 5% of the patients had major complication with no difference in the incidence between the two resection groups.

On average, the patients were overweight preoperatively defined by a BMI > 25 kg/m^2^ (left-sided resected patients: BMI 26.8, right-sided resected patients: BMI 25.6).

When stratifying the patients by sex, baseline characteristics were similar across the left-sided and right-sided resected men and women (Supplementary Tables 1 and 2), except that women in the right-sided resection group were older, had a lower cancer stage and received chemotherapy to a lesser extent than the left-sided resected women (Supplementary Table 2).

### Changes in VAT, SAT, TAT and V/S after 3 years follow-up within each resection group

**Fig. 3:**
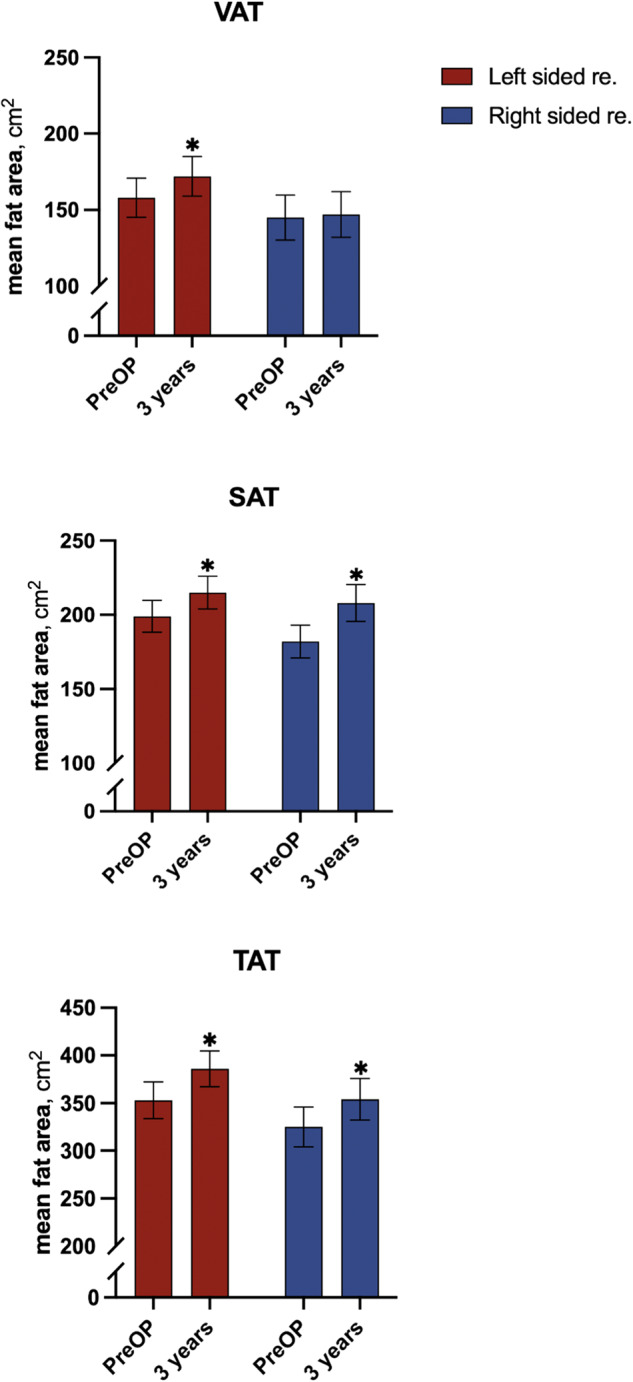
Changes in abdominal fat depots after left-sided and right-sided colonic resection in patients with colon cancer. Changes in visceral adipose tissue (VAT), subcutaneous adipose tissue (SAT) and total adipose tissue (TAT) measured in mean cm^2^ preoperatively (PreOP) to 3 years after cancer surgery with left-sided colonic resection (re) (red bars) and right-sided colonic resection (re) (blue bars). Data represent mean ± SEM. *p < 0.05 determined by a within group analysis from a linear mixed effect model.

After 3 years follow-up, left-sided resected survivors of CC had a 5% (*p* < 0.01) increase in VAT, a 4% (*p* < 0.001) increase in SAT and a 5% (*p* < 0.01) increase in TAT (Table 2 and Fig. 3). After 3 years follow-up, right-sided resected survivors of CC had no change in VAT, but a 6% (*p* < 0.001) increase in SAT and a 4% (*p* < 0.01) increase in TAT. Patients who had undergone left-sided resection had no change in V/S ratio, whereas V/S ratio decreased 4% (*p* = 0.02) after 3 years in the patients who had undergone right-sided resection.

**Table 2:** Changes in size of fat depots.

	Size of fat depots		In-group differences		Between-group differences			
	PreOP mean, cm^2^	3 years mean, cm^2^	PreOP vs. 3 years		PreOP		Change (PreOP-3 years)	
	±SEM	±SEM	Δ (95% CI)	*p*-value	Diff. (95% CI)	*p*-value	Diff. (95% CI)	*p*-value
All patients								
VAT, cm^2^								
Left-sided	158 ± 12.76	172 ± 12.92	1.05 (1.02–1.09)	<0.01	1.02 (0.97–1.09)	0.42	1.03 (0.98–1.09)	0.24
Right-sided	145 ± 14.78	147 ± 14.99	1.02 (0.98–1.06)	0.38				
SAT, cm^2^								
Left-sided	199 ± 10.75	215 ± 10.98	1.04 (1.02–1.06)	<0.001	1.06 (1.06–1.07)	0.87	0.98 (0.95–1.01)	0.24
Right-sided	182 ± 11.10	208 ± 12.38	1.06 (1.04–1.09)	<0.001				
TAT, cm^2^								
Left-sided	353 ± 19.26	386 ± 18.80	1.05 (1.02–1.07)	<0.01	1.02 (0.98–1.06)	0.77	1.00 (0.97–1.04)	0.92
Right-sided	325 ± 20.95	354 ± 21.68	1.04 (1.01–1.07)	<0.01				
V/S ratio								
Left-sided	0.85 ± 0.07	0.88 ± 0.07	1.01 (0.98–1.03)	0.59	1.05 (0.93–1.18)	0.41	1.05 (1.00–1.09)	0.03
Right-sided	0.80 ± 0.09	0.75 ± 0.09	0.96 (0.93–0.99)	0.02				
**Men**								
VAT, cm^2^								
Left-sided	206 ± 19.21	224 ± 18.89	1.06 (1.02–1.10)	<0.01	1.02 (0.94–1.11)	0.59	1.05 (0.98–1.13)	0.14
Right-sided	214 ± 25.95	212 ± 27.58	1.01 (0.95–1.06)	0.84				
SAT, cm^2^								
Left-sided	163 ± 11.62	176 ± 10.78	1.04 (1.01–1.07)	<0.01	0.99 (0.94–1.05)	0.85	0.99 (0.94–1.03)	0.52
Right-sided	152 ± 13.71	172 ± 16.47	1.06 (1.02–1.09)	<0.01				
TAT, cm^2^								
Left-sided	367 ± 29.18	398 ± 27.07	1.04 (1.01–1.08)	0.01	1.01 (0.94–1.08)	0.82	1.02 (0.96–1.07	0.66
Right-sided	366 ± 36.59	384 ± 38.80	1.03 (0.99–1.08)	0.14				
V/S ratio								
Left-sided	1.27 ± 0.09	1.27 ± 0.09	1.01 (0.98–1.04)	0.54	1.00 (0.89–1.13)	0.94	1.05 (1.01–1.10)	0.03
Right-sided	1.30 ± 0.15	1.21 ± 0.16	0.96 (0.93–0.99)	0.02				
**Women**								
VAT, cm^2^								
Left-sided	110 ± 13.17	119 ± 13.3	1.04 (0.99–1.10)	0.12	1.01 (0.93–1.10)	0.81	1.02 (0.94–1.10)	0.69
Right-sided	98 ± 11.86	101 ± 11.50	1.03 (0.97–1.09)	0.37				
SAT, cm^2^								
Left-sided	234 ± 16.34	257 ± 17.12	1.05 (1.01–1.08)	<0.01	1.00 (0.96–1.05)	0.83	0.98 (0.93–1.03)	0.38
Right-sided	205 ± 15.38	236 ± 16.28	1.07 (1.03–1.11)	<0.001				
TAT, cm^2^								
Left-sided	339 ± 25.39	373 ± 26.27	1.05 (1.01–1.08)	<0.01	1.01 (0.96–1.06)	0.78	0.99 (0.94–1.05)	0.79
Right-sided	292 ± 23.48	332 ± 24.55	1.05 (1.01–1.10)	0.01				
V/S ratio								
Left-sided	0.45 ± 0.05	0.46 ± 0.05	1.01 (0.96–1.05)	0.79	1.00 (0.89–1.13)	0.99	1.04 (0.97–1.12)	0.27
Right-sided	0.41 ± 0.04	0.40 ± 0.04	0.97 (0.92–1.02)	0.23				

Means (SEM) are based on available data (PreOp, 3 years) for patients who underwent left-sided or right-sided colonic cancer resection.

In-group and between-group differences were analyzed using a unified linear mixed effects model. Logarithmic values were used. In group and between group changes don’t reflect the numerical difference between preoperative and 3 years, given that mean change is estimated based on mixed-model analysis.

*VAT* Visceral adipose tissue, *SAT* Subcutaneous adipose tissue, *TAT* Total adipose tissue, *V/S* VAT/SAT ratio, *PreOP* Preoperative.

### Differences in VAT, SAT, TAT and V/S between the resection groups

Preoperatively no difference was found between patients who had undergone left-sided and right-sided resection (Table 2). After 3-years follow-up, we found no difference in the mean changes in SAT, TAT and V/S ratio between left-sided and right-sided resection group. Moreover, when evaluating VAT in the main analysis, the group x time interaction was insignificant (*p* = 0.24).

### Within group changes in VAT, SAT, TAT and V/S in men and women separately

Within group changes in VAT, SAT, TAT and V/S for men and women separately are presented in supplementary Table 1. After 3-years follow up, left-sided resected men had an increased in VAT (6%, *p* < 0.01), SAT (4%, *p* < 0.01) and TAT (5%, *p* < 0.01), whereas women had an increased SAT (5%, *p* < 0.01) and TAT (5%, *p* < 0.01), but not VAT.

For both men and women, who had undergone right-sided cancer resection, no changes in VAT were observed after 3 years. In men a 6% (*p* < 0.01) increase in SAT and no change in TAT was observed after 3 years. In right-sided resected women a 7% (*p* < 0.001) increase in SAT and a 5% (*p* = 0.01) increase in TAT was observed after 3 years.

In men, the V/S ratio in the left-sided resected group was unchanged after 3 years follow-up, while it was decreased 4% (*p* = 0.02) in right-sided resected. In women, the V/S-ratio did not change over time in any of the groups.

### Differences in VAT, SAT, TAT and V/S between resection groups in men and women separately

Stratifying by gender did not change the results; preoperative measurement as well as the mean changes after 3 years follow-up in SAT, TAT and V/S ratio were similar in patients who had undergone left-sided and right-sided resection. Between group differences in men and women separately are presented in supplementary Table 1. When comparing changes in SAT, TAT and V/S ratio within the two resection groups preoperatively to 3 years after surgery we found no significant difference between the two resection groups in either men or women. When comparing VAT, SAT, and TAT in patients who had undergone right-sided vs. left-sided resection preoperatively no difference was found in either men or women.

### Diabetes development

After reviewing medical records, none of the patients were diagnosed with T2D or were prescribed anti-diabetic medication during 3 years of surveillance. Thus, it was not possible to assess changes in diabetes development across the groups.

## Discussion

Our main finding was that patients operated for stage I-III CC increase in abdominal adiposity over the course of 3 years following cancer surgery, but there may be a tendency towards higher VAT accumulation in patients undergoing left-sided colonic resection. This trend was similar for both men and women, but only statistically significant for male survivors of CC.

Due to the potential prognostic significance of increased VAT in left-sided resected survivors of CC may constitute a population at risk of developing metabolic disturbances and having an inferior cancer prognosis.

The overall increase in VAT after surgical cancer treatment is in line with our prior findings [26]. In that study it was speculated whether the type of cancer resection per se has the potential to alter lipid metabolism.

Our data indicate that left-sided colonic resected survivors of CC may constitute a risk population. This is in line with two recent epidemiological studies. In 2018 Jensen and coworkers showed that left-sided colonic resections compared with right-sided colonic resections was associated with an increased risk of T2D in patients with CC and non-cancer patients suffering from inflammatory bowel disease etc. [27]. Later, a study from Taiwan explored the association between resection type and metabolic disturbances in patients without CC and found a higher CVD risk after left-sided resection, and a reduction in T2D after right-sided resection when compared with non-colectomy subjects [38]. However, the most recent epidemiological study found an increased risk of T2D after colectomy compared with small bowel resection, but when stratifying by resection type, no difference in T2D risk was found [39].

According to a prior study establishing a cut-off value of pathological obesity in patients with cancer, a VAT of 163.8 cm^2^ in males (83.6% sensitivity, 62.5% specificity) and 80.1 cm^2^ in females (96% sensitivity, 73.2% specificity) was strongly correlated with presence of the metabolic syndrome, a precursor of T2D, in Caucasians [40]. Accordingly, based on these cut-off values both males and females independent of resection type had pathological abdominal obesity preoperatively in the present study, which worsened during the follow up period. Based on VAT data it is likely that some of the patients may have developed metabolic syndrome 3 years after cancer surgery, but we didn’t have the relevant variables to detect it post-surgery.

It is well known that excess VAT is accompanied by low-grade inflammatory changes within the fat depot. This contributes to chronic systemic inflammation with enhanced concentrations of circulating cytokines such as CRP and IL-6 and adipokines [41], which is associated with cancer progression and poor survival in patients with CC [42]. In the present study CRP and IL-6 levels were only available preoperatively and values were within the normal range in both resection groups and in men and women respectively.

Our data revealed that SAT was increased independent of resection type and in both sexes after 3 years. In contrast to VAT, the role of abdominal SAT in development of diabetes, CVD and cancer recurrence and death after cancer resection is not fully understood [43, 44]. Nonetheless, an increase in VAT is highly correlated with an increase in SAT [43, 44].

Until recently the impact of adiposity on CC prognosis has been confusing [45]. The divergent results may be due to the various ways to measure adiposity in former studies (eg BMI, waist-circumference, bioelectrical impedance, quantitative CT-scans etc.) [46]. However, since excess adipose tissue is believed to be involved in the underlying pathogenesis of CC [47], it seems plausible that adiposity is associated with more aggressive cancers with increased rates of recurrence. This was confirmed in a recent and the largest metanalysis including 45 studies and 607,266 patients with stage I to IV colorectal cancer as they reported a significant increase in cancer specific (OR = 1.27; 95% CI 1.11–1.45) and overall (OR = 1.20; 95% CI 1.06–1.36) mortality in obese patients compared with patients who were normal-weight [45]. Moreover, increased waist circumference, which is strongly correlated to VAT [48], was associated with increased cancer specific mortality [45].

The distribution of abdominal fat expressed as the V/S ratio has been used as prognostic marker in prior studies. In a study by Hyeong-Gon Moon et al. including 161 patients who had undergone colonic resection for CRC (both men and women) revealed that a preoperative V/S ratio > 0.83 (average V/S-ratio preoperatively was 0.83 in our study) was associated with significantly lower cumulative disease-free survival rate during 8 years of post-operative follow-up [21]. Based on this study survivors of CC who had undergone left-sided colonic resection in the present study may have a worse prognosis. However, this is in contrast to a prospective observational study showing that overall survival was reduced in right-sided compared with left-sided resected patients with CRC [49], and similar studies suggest that tumor location have no impact on overall survival [50].

Due to the strong association between excess VAT and morbidity and mortality in survivors of CC, identifying survivors of CC with increased amounts of VAT may be of great clinical relevance. Our study revealed a tendency towards increased VAT after left-sided CC resection. This is in line with two prior epidemiological studies showing that patients who had undergone left-sided colonic resection may constitute a risk population.

A strength of the present work is that the method used as quantitative computed tomography (CT) to determine VAT, SAT and TAT in CC is well established. CT scans were consistently performed before surgery in relation to cancer staging and later until 3 years postoperatively to detect disease recurrence. Furthermore, all analyses were performed blinded to type of surgery (right or left-sided resection) and were performed by two independent investigators. Another strength of this study is the prospective nature of the study allowing us to detect changes in abdominal fat depots over time.

Our study also has several limitations listed below. First of all, we lack all other variables except for CT-scan results after 3 years. In addition, due to the exploratory nature of the study there is a risk of type 2 statistical errors for some parameters including the lack of difference in the between group comparisons for VAT in opposition to the clear increase in VAT in the left-sided resected but not the right sided resected in the with-in group analysis.

Another limitation is the difference in administration of adjuvant chemotherapy between resection groups. When comparing baseline characteristics, the left-sided tumors were detected at a slightly more advanced cancer stage, and therefore adjuvant chemotherapy was administered more frequent after left-sided resections. However, to the best of our knowledge there is no evidence suggesting that 3–6 months of adjuvant chemotherapy treatment with FOLFOX, CAPOX or monotherapy 5-FU or capecitabine postoperatively affects changes in abdominal adipose tissue after 3 years.

To avoid adverse effects during each cycle of adjuvant chemotherapy some patients receive a few days days of prednisolone treatment [31]. Thus, in this study patients who had undergone left-sided resection may have received larger amounts of prednisolone well known to induce hyperglycemia [51] and central obesity [52]. Unfortunately, we do not have information regarding the amount of prednisolone each patient received during treatment with adjuvant chemotherapy. However, as prednisolone treatment will typically only be administered for a few days each month when a new cycle of chemotherapy is initiated, we do not expect the applied doses of prednisolone to have affected metabolism over a time course of 3 years in this study [53]. Finally, weight loss prior to cancer surgery was not recorded systematically.

.

## Conclusion

After 3 years follow-up survivors of CC accumulated abdominal adipose tissue. Notably, those who underwent left-sided colonic resection had increased VAT and SAT, whereas patients who had undergone right-sided colonic resection demonstrated solely increased SAT.

### Supplementary information

Suplemental_Table 2

Suplemental_Table 1

## Data Availability

The data that support the findings of this study are available from [louise.lang.lehrskov.01@regionh.dk] but restrictions apply to the availability of these data, which were used under license for the current study, and so are not publicly available. Data are however available from the authors upon reasonable request and with permission of [louise.lang.lehrskov.01@regionh.dk].
