# Supplementary material for: Change in abdominal obesity after colon cancer surgery – effects of left-sided and right-sided colonic resection
Source: Int J Obes (Lond). 2024 Jan 3;48(4):533–41. doi: 10.1038/s41366-023-01445-8 (PMC10978490; doi:10.1038/s41366-023-01445-8)
Supplement: Supplementary file 1 — Suplemental_Table 2 [file 41366_2023_1445_MOESM1_ESM.docx]

| **SUPLEMENTARY TABLE 2: Baseline characteristics for women** | | | | |
| --- | --- | --- | --- | --- |
|  | **All**  ***n* = 72** | **Left sided resection**  ***n* = 40** | **Right sided resection**  ***n* = 32** | **p-value** |
| **Age**, year, mean (SD) | 67 (9.5) | 64 (9.5) | 70 (8.2) | <0.01 |
| **Overall stage**, n (%) |  |  |  |  |
| 1 | 23 (31.9) | 12 (30.0) | 11 (34.4) | 0.70 |
| 2a | 27 (37.5) | 13 (32.5) | 14 (43.8) | 0.34 |
| 2b | 1 (1.4) | 1 (2.5) | 0 (0.0) | 0.32 |
| 3a | 8 (11.1) | 4 (10.0) | 4 (12.5) | 0.74 |
| 3b | 13 (18.1) | 10 (25.0) | 3 (9.4) | 0.08 |
| **ECOG performance status^1^**, n (%) | |  |  |  |
| 0 | 62 (86.1) | 37 (92.5) | 25 (78.1) | 0.10 |
| 1 | 7 (9.7) | 3 (7.5) | 4 (12.5) | 0.50 |
| 2 | 3 (4.2) | 0 (0.0) | 3 (9.4) | 0.08 |
| **Anthropometric indices**, mean (SD) | |  |  |  |
| Weight, kg | 71.8 (13.9) | 74.1 (16.0) | 68.9 (10.3) | 0.11 |
| Height, cm | 165.6 (5.8) | 165.2 (5.9) | 166.1 (5.8) | 0.52 |
| BMI, kg/m^2^ | 26.2 (5.1) | 27.1 (5.6) | 25.1 (4.2) | 0.08 |
| **Smoking**, n (%) |  |  |  |  |
| Never | 40 (55.6) | 25 (62.5) | 15 (46.9) | 0.19 |
| Former | 22 (30.6) | 11 (27.5) | 11 (34.4) | 0.54 |
| Current | 8 (11.1) | 2 (5.0) | 6 (18.8) | 0.09 |
| Not known | 2 (2.8) | 2 (5.0) | 0 (0.0) | 0.16 |
| **Alcohol**, n (%) |  |  |  |  |
| Never | 22 (30.6) | 14 (35.0) | 8 (25.0) | 0.36 |
| Normal | 39 (54.2) | 19 (47.5) | 20 (62.5) | 0.21 |
| Overuse | 9 (12.5) | 5 (12.5) | 4 (12.5) | 1.00 |
| Previous overuse | 0 (0.0) | 0 (0.0) | 0 (0.0) | NA |
| Not known | 2 (2.8) | 2 (5.0) | 0 (0.0) | 0.16 |
| **Comorbidities**, n (%) |  |  |  |  |
| Hypertension | 20 (27.8) | 11 (27.5) | 9 (28.1) | 0.95 |
| Hyperlipidemia | 11 (15.3) | 5 (12.5) | 6 (18.8) | 0.48 |
| COPD | 6 (8.3) | 2 (5.0) | 4 (12.5) | 0.28 |
| Cardiovascular disease | 2 (2.8) | 1 (2.5) | 1 (3.1) | 0.88 |
| Others | 2 (2.8) | 2 (5.0) | 0 (0.0) | 0.16 |
| **Pharmacological treatment**, n (%) | |  |  |  |
| Prednisolone | 2 (2.8) | 1 (2.5) | 1 (3.1) | 0.88 |
| Antihypertensive medication | 47 (65.3) | 25 (62.5) | 22 (68.8) | 0.58 |
| **Biochemical values**, median (range) | |  |  |  |
| IL6, ng/L | 2.2 (0.62-42.8) | 2.0 (0.62-32.8) | 2.4 (1.1-42.8) | 0.46 |
| CRP, mg/L | 1.2 (0.0-76.0) | 0.62 (0.0-76.0) | 1.9 (0.0-51.9) | 0.47 |

IL-6, Interleukin-6; CRP, C-reactive protein. An unpaired *t*-test was used to calculate *P* values*.*

^1.^ECOG performance status definitions: 0: Fully active, able to carry on all pre-disease performance without restriction; 1: Restricted in physically strenuous activity but ambulatory and able to carry out work of a light or sedentary nature, e.g., light housework, office work; and 2: Ambulatory and capable of all selfcare but unable to carry out any work activities; up and about more than 50% of waking hours (53).
