# Supplementary material for: Change in abdominal obesity after colon cancer surgery – effects of left-sided and right-sided colonic resection
Source: Int J Obes (Lond). 2024 Jan 3;48(4):533–41. doi: 10.1038/s41366-023-01445-8 (PMC10978490; doi:10.1038/s41366-023-01445-8)
Supplement: Supplementary file 2 — Suplemental_Table 1 [file 41366_2023_1445_MOESM2_ESM.docx]

| **SUPPLEMENTARY TABLE 1: Baseline characteristics for men** | | | | |
| --- | --- | --- | --- | --- |
|  | **All**  ***n* = 62** | **Left sided resection**  ***n* = 40** | **Right sided resection**  ***n* = 22** | **p-value** |
| **Age**, year, mean (SD) | 67 (8.5) | 67 (7.7) | 67 (10.0) | 0.96 |
| **Overall stage**, n (%) |  |  |  |  |
| 1 | 15 (24.2) | 9 (22.5) | 6 (27.3) | 0.69 |
| 2a | 24 (38.7) | 16 (40.0) | 8 (36.4) | 0.78 |
| 2b | 4 (6.5) | 2 (5.0) | 2 (9.1) | 0.57 |
| 3a | 4 (6.5) | 1 (2.5) | 3 (13.6) | 0.17 |
| 3b | 15 (24.2) | 12 (30.0) | 3 (13.6) | 0.12 |
| **ECOG performance status^1^**, n (%) | |  |  |  |
| 0 | 49 (79.0) | 33 (82.5) | 16 (72.7) | 0.40 |
| 1 | 10 (16.1) | 6 (15.0) | 4 (18.2) | 0.76 |
| 2 | 3 (4.8) | 1 (2.5) | 2 (9.1) | 0.34 |
| **Anthropometric indices**, mean (SD) | |  |  |  |
| Weight, kg | 86.1 (14.1) | 85.2 (13.6) | 87.7 (15.2) | 0.54 |
| Height, cm | 180.4 (7.0) | 179.6 (7.2) | 181.9 (6.6) | 0.22 |
| BMI, kg/m^2^ | 26.4 (3.9) | 26.4 (3.8) | 26.5 (4.1) | 0.95 |
| **Smoking**, n (%) |  |  |  |  |
| Never | 24 (38.7) | 15 (37.5) | 9 (40.9) | 0.80 |
| Former | 29 (46.8) | 20 (50.0) | 9 (40.9) | 0.50 |
| Current | 6 (9.7) | 3 (7.5) | 3 (13.6) | 0.48 |
| Not known | 3 (4.8) | 2 (5.0) | 1 (4.5) | 0.70 |
| **Alcohol**, n (%) |  |  |  |  |
| Never | 10 (16.1) | 7 (17.5) | 3 (13.6) | 0.69 |
| Normal | 39 (62.9) | 26 (65.0) | 13 (59.1) | 0.66 |
| Overuse | 11 (17.7) | 6 (15.0) | 5 (22.7) | 0.48 |
| Previous overuse | 1 (1.6) | 1 (2.5) | 0 (0.0) | 0.32 |
| Not known | 1 (1.6) | 0 (0.0) | 1 (4.5) | 0.33 |
| **Comorbidities**, n (%) |  |  |  |  |
| Hypertension | 24 (38.7) | 14 (35.0) | 10 (45.5) | 0.44 |
| Hyperlipidemia | 18 (29.0) | 11 (27.5) | 7 (31.8) | 0.73 |
| COPD | 4 (6.5) | 3 (7.5) | 1 (4.5) | 0.64 |
| Cardiovascular disease | 6 (9.7) | 3 (7.5) | 3 (13.6) | 0.48 |
| Others | 1 (1.6) | 0 (0.0) | 0 (0.0) | 0.33 |
| **Pharmacological treatment**, n (%) | |  |  |  |
| Prednisolone | 2 (3.2) | 1 (2.5) | 1 (4.5) | 0.69 |
| Antihypertensive medication | 42 (67.7) | 26 (65.0) | 16 (72.7) | 0.54 |
| **Biochemical values**, median (range) | |  |  |  |
| IL6, ng/L | 2.3 (0.6-71.9) | 2.0 (0.6-59.6) | 2.4 (0.9-71.9) | 0.67 |
| CRP, mg/L | 0.7 (0.0-76.0) | 1.2 (0.0-76.0) | 0.0 (0.0-24.0) | 0.34 |

IL-6, Interleukin-6; CRP, C-reactive protein. An unpaired *t*-test was used to calculate *P* values*.*

^1.^ECOG performance status definitions: 0: Fully active, able to carry on all pre-disease performance without restriction; 1: Restricted in physically strenuous activity but ambulatory and able to carry out work of a light or sedentary nature, e.g., light housework, office work; and 2: Ambulatory and capable of all selfcare but unable to carry out any work activities; up and about more than 50% of waking hours (53).
